# Obstetric Healthcare Workers’ Adherence to Hand Hygiene Recommendations during the COVID‐19 Pandemic: Observations and Social‐Cognitive Determinants

**DOI:** 10.1111/aphw.12240

**Published:** 2020-10-05

**Authors:** Christina Derksen, Franziska M. Keller, Sonia Lippke

**Affiliations:** ^1^ Jacobs University Bremen gGmbH Germany

**Keywords:** COVID‐19, hand hygiene behaviour, health action process approach, observations, obstetrics and gynaecology, social‐cognitive determinants

## Abstract

**Background:**

Hand hygiene is crucial to avoid healthcare‐associated infections and the transmission of COVID‐19. Although the WHO has issued global hand hygiene recommendations for healthcare, adherence remains challenging. Considering social‐cognitive theories such as the *health action process approach* (HAPA) can help to improve healthcare workers’ adherence. This study aimed to observe adherence and to assess determinants in obstetric hospitals during and after the onset of the COVID‐19 pandemic.

**Methods:**

In all, 267 observations of behaviour were conducted in two German obstetric university hospitals over three time periods (*pre‐COVID‐19 pandemic*, *heightened awareness*, and *strict precautions*). In addition, 115 healthcare workers answered questionnaires regarding social‐cognitive determinants of hand hygiene behaviour. Multiple regression and multiple mediation analyses were used to analyse associations.

**Results:**

Adherence to hand hygiene recommendations increased from 47 per cent *pre‐COVID‐19 pandemic* to 95 per cent just before lockdown while simple measures against the pandemic were taken. Self‐efficacy was associated with the intention to sanitise hands (β = .397, *p* < .001). Coping self‐efficacy mediated the association of intention with hand hygiene adherence.

**Conclusions:**

Obstetric healthcare workers seem to adapt their hand hygiene behaviour to prevent infections facing the global COVID‐19 pandemic. To further improve interventions, social‐cognitive determinants should be considered, especially intention and (coping) self‐efficacy.

## INTRODUCTION

Thorough hand hygiene is the most important method to avoid *healthcare‐associated infections* (HAI; Sickbert‐Bennett et al., 2016). HAI are among the most prevalent (preventable) adverse events (pAE) in healthcare, having a severely negative effect on patient outcomes and thus patient safety. In 2016, Cassini et al. estimated that more than 2.5 million new cases of the six most common HAI occur each year in the European Union (EU). Taken together, these infections cause a burden of 501 disability‐adjusted life years (DALYs) per 100,000 general population. This is among the highest disease burdens of communicable diseases and illustrates the need to prevent HAI worldwide (Cassini et al., 2016). *Adherence to hand hygiene recommendations* has gained even more significance since the beginning of the COVID‐19 pandemic. The 2019 novel coronavirus (SARS‐CoV‐2) is transmitted mostly via projection of aerosols, but can be transmitted by physical contact or contact with infected surfaces and material as well (Asadi et al., 2020). To manage the pandemic, different actions need to be taken to stop its spreading. Since hospitals are a crucial source of infections, appropriate actions concerning hand hygiene need to be implemented according to the World Health Organization (WHO, 2020). It is yet unclear whether hand hygiene behaviour changed over the progression of the pandemic or to what extent the COVID‐19 pandemic influenced social‐cognitive determinants of behaviour such as attitudes and perceptions.

The WHO had issued *global guidelines* and recommendations for hand hygiene in healthcare long before the start of the COVID‐19 pandemic. They identify *five moments when to wash or sanitise hands* in clinical routines which include (1) *before touching a patient* and (2) *after touching a patient*, (3) *before aseptic procedures*, (4) *after touching patient surroundings*, and (5) *after body fluid exposure risk* (WHO Patient Safety Alliance, 2009). These recommendations have been applied in different countries worldwide (Sax et al., 2017).

A main cause of HAI is the lack of adherence to these standards (WHO Patient Safety Alliance, 2009). *Measuring adherence* to hand hygiene recommendations is challenging, and there are different approaches from low to high technology. Although automated hand hygiene monitoring systems seem promising to enhance adherence to hand hygiene recommendations, they require more financial and human resources and might be perceived as intrusive by healthcare workers (HCW; Masroor et al., 2017). However, self‐reports of hand hygiene behaviour have been found to be unreliable in several studies when compared to standardised observations (Alshammari et al., 2018; Jenner et al., 2006). Social desirability accounts for a substantial part of answering biases, but dissonance and encoding/decoding processes also explain why self‐reports are insufficient (Contzen, Pasquale, & Mosler, 2015). Additionally, self‐serving biases can lead to an overestimation of one’s adherence to hand hygiene recommendations (Foà et al., 2017). Thus, observation by trained personnel is currently considered the gold standard for hand hygiene measures, if possible in combination with product usage monitoring to ensure measurement objectivity (Boyce, 2008). Observed adherence to hand hygiene recommendations has been found to vary from 5 per cent to 89 per cent, with an overall average of 38.7 per cent (WHO Patient Safety Alliance, 2009). Especially in *obstetrics*, hand hygiene adherence is often poor (Cantrell et al., 2009; Santosaningsih et al., 2017). However, giving birth can leave mothers and children vulnerable for postpartum infections. Today, standards are put in place for all areas of healthcare, but adherence to the regulations and hand hygiene recommendations remain a relentless challenge (Boyce, 2019).

In Germany, the Coalition for Patient Safety adapted the WHO guidelines and started an initiative called “Aktion Saubere Hände” (“Clean Hands Campaign”) to prevent up to 150,000 infections per year (Reichardt et al., 2008). The first results have shown a positive effect of the campaign on sanitiser use and reduction of infections; nevertheless, achieving compliance continues to be problematic (Reichardt et al., 2014). Identified *hindering factors* are time pressure and a high workload (Houghton et al., 2020), but attitudes, social support, and intention to wash hands frequently can be important as well (Pessoa‐Silva et al., 2005). In a systematic qualitative review, Smiddy et al. (2015) identified crucial motivational factors such as social influences, emergencies, and use of cues. The work environment including resources, knowledge, and organisational culture also influenced adherence (Smiddy et al., 2015). Resources that can facilitate hand hygiene behaviour are availability of dispensers and other disinfectants, information and training seminars, reminders, and social support (Sadule‐Rios & Aguilera, 2017). All of these factors may be associated with hand hygiene behaviour even in the time of the COVID‐19 pandemic.

Given these social determinants, adherence to hand hygiene can be explained with regard to *social‐cognitive model*s considering motivational factors and perception of resources. Especially in the recent years, promising hand hygiene interventions have been based on psychological frameworks of behaviour change, even outside of the clinical context (Contzen, Meili, & Mosler, 2015; Reyes Fernández et al., 2016). In the clinical context, Srigley et al. (2015) only identified three studies in which social‐cognitive theories were specifically used to predict hand hygiene behaviour as well as four intervention studies that aimed to change hand hygiene behaviour among HCW based on explicitly named psychological theories in 2015. The results were mixed: Hand hygiene behaviour appears to be unrelated to attitudes and intention, but associated with decision‐making and knowledge as well as stages of change. Thus, it remains rather unclear which social‐cognitive processes determine effective hand hygiene behaviour, and theoretical models need to be applied.

The *health action process approach* (HAPA; Schwarzer, 2008) integrates and advances previous health behaviour change theories. The HAPA model distinguishes behaviour change in two phases: in the motivational phase, *risk perceptions* (i.e. feeling at risk when handwashing is not carried out appropriately) start a contemplation process to form an *intention to change a specific behaviour* while *outcome expectancies* (i.e. expecting that changing to better hand hygiene has certain outcomes) as well as *self‐efficacy* (i.e. being confident in one’s ability to change to better hand hygiene) help to form this intention more proximately (Lippke et al., 2010). In the volitional phase, the gap between an intention and actually enacting the respective behaviour is bridged by *action and coping planning* (i.e. planning one’s own behaviour in detail even if a barrier occurs; Schwarzer et al., 2011). Maintaining self‐efficacy also helps to mediate between intention and behaviour (Sniehotta et al., 2005). Since coping plays an important role during the pandemic and self‐efficacy can facilitate behaviour, we will investigate coping self‐efficacy as a possible mediator synonymously with maintenance self‐efficacy.

Recently, researchers have used the HAPA model to inform an *intervention to enhance hand hygiene behaviour* in intensive care units with promising results, showing that psychological interventions tailored to unit characteristics increased hand hygiene adherence long‐term and thus reduced HAI (Lengerke et al., 2019; Lengerke et al., 2017). However, they did not measure which predictors influenced self‐reported hand hygiene behaviour directly in clinical practice (Porst et al., 2012).

Therefore, we aim to investigate two objectives with this paper. First, we aim to examine to what extent the COVID‐19 pandemic is related to hand hygiene behaviour among obstetricians and midwives, and second, which social‐cognitive factors determine their hand hygiene behaviour. To test if self‐reported adherence among obstetric employees was accurate, we aim to compare self‐reported behaviour to observed adherence. Our hypotheses are:

(1) *Observed adherence* to hand hygiene recommendations improves after the outbreak of the COVID‐19 pandemic in Germany. (2) Social‐cognitive factors explain hand hygiene behaviour. This includes forming an *intention* and showing according to *behaviour*: (2a) Self‐efficacy, risk perceptions, and outcome expectancies are associated with the *intention* to wash and sanitise hands according to WHO standards. (2b) Intention to wash and sanitise hands is associated with self‐reported hand hygiene *behaviour*, mediated by coping self‐efficacy, coping planning, and perceived resources.

## METHODS

### Setting

This study was conducted as part of the research project “TeamBaby—Communication and patient safety in gynecology and obstetrics” (ClinicalTrials.gov Identifier: NCT03855735). Data were collected in two obstetric university hospitals in Germany. Both hospitals had large perinatal clinics providing the highest level of care with approximately 2,800 to 3,200 deliveries every year and affiliated neonatal intensive care units (NICU). About 50 per cent of deliveries were medium‐to‐high risk.

Data were collected from 2 January to 15 March 2020. The data collection period included a “pre‐COVID‐19 pandemic period” in January (the first COVID‐19 case in Germany was registered on 28 January), a period of “heightened awareness” in which the first measures were taken such as displaying posters as well as setting up more disinfectant dispensers (February), and a period of “strict precautions” in which standard operational procedures regarding COVID‐19 were implemented via email and personal meetings (24 February to 15 March). Operational procedures included testing and isolating patients who scored >0 on a COVID‐19 symptom questionnaire, wearing face masks at all times, and taking special precautions during delivery. In one hospital, fathers were still allowed into the delivery rooms, which was not the case in the second hospital. On 16 March, researchers were prohibited from accessing the delivery rooms and postpartum units due to COVID‐19 regulations issued by the local health authorities. Only HCW who were crucial for patient care and safety were granted access to the hospitals. An overview of data collection is provided in the supplementary materials (Table S1).

### Recruitment and Procedure

At both hospitals, a research associate and a study nurse recruited participants. Affiliated personnel from both hospitals (e.g. the assistant medical directors, senior consultants, and head midwives) helped the recruitment in team meetings and via personal contact. Departments of quality management were involved to ensure adequate participant enrollment and to avoid a selection bias. HCW were informed about the research project in group meetings and were handed contact details of the on‐site researchers in case they had additional questions. They were given additional written information and informed consent forms.

### Observations

Eligible participants for the observations were HCW (physicians, midwives, and nurses) in the two obstetric university hospitals that are part of the research project “TeamBaby”. Trainees were included as well as HCW occupying a higher role at either the labour and delivery units or the postpartum care units. The aim was to observe all HCW who worked at least part‐time in any obstetric unit or in a gynaecological unit which was closely affiliated with the delivery rooms (*n *=* *140). First non‐participatory observations were conducted in the first university hospital from 21 January until 2 February (*pre‐COVID‐19 pandemic period*) and in the other university hospital from 18 to 21 February (*heightened awareness period*). In the first hospital, more observations were conducted with the same HCW who were observed in the first time period from 9 to 13 March (shortly before lockdown, *period of strict precautions*) to control for influences of the COVID‐19 pandemic. Observations were conducted with an emphasis on the *pre‐COVID‐19 pandemic* (148 observations) and *heightened awareness* period (98 observations). In the third period, only 21 moments could be observed before the lockdown on 16 March.

Healthcare workers were informed that their communication, teamwork, and adherence to patient safety recommendations including hand hygiene behaviour were going to be observed (data on communication and teamwork will be published elsewhere). Researchers used the German adaptation of the WHO gold standard, namely “Clean Hands Campaign” observation sheets (see Appendix S1 for further description). The observers were asked to position themselves in the corners of the delivery rooms or to walk behind HCW to be less obtrusive. Observation periods ranged from 1 to 2.5 hr. HCW knew that their communication, teamwork, and hand hygiene behaviour were observed but they did not know which one was being observed at any specific observation period. In each observation, up to three HCW were observed simultaneously when possible. However, the researchers started observing different HCW in the same observation period if a HCW was unlikely to have moments to wash their hands within the next few minutes (e.g. when documenting). Thus, approximately 3–10 HCW were observed during an observation period. There were 19 observation periods (two in the *strict precautions* time period).

#### Analysis of Observations

Observations are presented as total numbers and percentages of adherent reactions to moments to wash or sanitise hands. Differences between the two hospitals’ observed time periods were tested without control variables via χ^2^‐tests if the cell count was >5. A χ^2^‐test was also conducted to test for differences between occupational groups. The observations from the first two time periods were conducted in different hospitals.

### Self‐reported Data

Participants who were eligible for observation were asked to provide questionnaire data independently from the observations. They were occasionally reminded to fill in the questionnaire via personal contact, email, or WhatsApp messages from the head midwife and short notes. *N* = 115 HCW provided self‐reported data concerning hand hygiene behaviour and its social‐cognitive determinants. Of all HCW, *n* = 53 came from the first hospital and *n* = 62 from the second. A detailed overview of socio‐demographic data is provided in Table 1.

**TABLE 1:** Overview of Socio‐Demographic Data and Experience among Health Care Providers

	*N = 115*	*Physicians (n = 44, 38%)*	*Midwives (n = 38, 33%)*	*Nurses (n = 12, 10%)*	*Trainees (to become nurses or a midwives) (n = 10, 9%)*	*Other (specified, e.g. medical assistant, and unspecified) (n = 11, 10%)*
Sex	Women (*n* = 105, 91%)	38 (86%)	38 (100%)	12 (100%)	9 (90%)	8 (73%)
	Men (*n* = 8, 7%)	5 (11%)	0 (0%)	0 (0%)	1 (10%)	2 (18%)
Age	<26 years (*n* = 24, 21%)	0 (0%)	11 (29%)	2 (17%)	10 (100%)	1 (9%)
	26–40 years (*n* = 63, 55%)	36 (82%)	17 (45%)	5 (42%)	0 (0%)	5 (45%)
	>40 years (*n* = 23, 20%)	5 (11%)	9 (24%)	5 (42%)	0 (0%)	4 (36%)
Experience	<1 year (*n* = 14, 12%)	4 (9%)	4 (11%)	0 (0%)	5 (50%)	1 (9%)
	1–5 years (*n* = 47, 41%)	18 (41%)	19 (50%)	2 (17%)	5 (50%)	3 (27%)
	>5 years (*n* = 48, 42%)	20 (45%)	14 (37%)	9 (75%)	0 (0%)	5 (45%)

Note

Frequencies and percentages are shown for each occupational group. Up to 6 participants did not provide information on sex, age, and/or level of experience.

Paper‐and‐pencil questionnaires were collected during the first two time periods at both hospitals simultaneously (*n* = 77 in the *pre‐COVID‐19 pandemic* and *n* = 21 in the *heightened awareness period*;*n *=* *17 questionnaires were unclear since participants did not provide a date on their informed consent form). The HAPA hand hygiene questionnaire was an adapted version of the “PSYGIENE” questionnaire developed for a tailored intervention study by von Lengerke et al. (Lengerke et al., 2017; Porst et al., 2012). The questionnaire was adapted to the five WHO moments so that observations and self‐reported data could be compared in greater detail. Intention and motivation scales were split according to the WHO moments to wash or sanitise one’s hands. A self‐constructed behaviour scale for self‐reported adherence to hand hygiene moments was added.

The questionnaire included short scales regarding intention (e.g. “I intend to wash my hands before aseptic procedures”, five items, Cronbach’s*α *= .65), positive and negative outcome expectancies (e.g. “If I wash or sanitise my hands before and after each contact with a patient, I’ll reduce the risk for healthcare‐associated infections”, positive: five items, Cronbach’s α* *= .68; negative: three items, Cronbach’s*α *= .73), coping planning (e.g. “I’ve planned how to maintain good hand hygiene even if I forgot hand disinfection in the first place”, three items, Cronbach’s*α *= .82), coping self‐efficacy (e.g. “I’m confident that I’ll manage to wash or sanitise my hands even if it takes time to adjust”, three items, Cronbach’s *α *= .81), hand hygiene behaviour (e.g. “I wash or sanitise my hands after body fluid exposure risk”, five items, Cronbach’s*α* = .73), and perceived resources (e.g. “I get a lot of information and training about good hand hygiene”, three items, Cronbach’s*α *= .79). Self‐efficacy was assessed with a single‐item scale (“I’m confident that I’ll manage to wash or sanitise my hands before and after each contact with a patient”), as was risk perception (“How high do you think is the probability that you’ll spread healthcare‐associated infections?”). All items were measured on a 6‐point Likert scale (1 = “Absolutely not” to 6 = “Absolutely”) except for risk perception which was measured on a 5‐point Likert scale (1 = “Highly unlikely” to 5 = “Highly likely”).

Furthermore, the questionnaire included socio‐demographic questions regarding sex, age, and profession. All were assessed as categorical data due to the requirements of data security of the quality management department. In particular, age needed to be assessed in four categories to ensure that on‐site researchers could not identify participants based on their demographic data in the questionnaire. There was always the option “I’d rather not say” if participants were not comfortable with one or more of the socio‐demographic questions. Age and profession were categorised into four groups each (“younger than or 25 years old”, “26–40 years old”, “41–55 years old”, “56 years old or older”, and “physician”, “midwife”, “nurse”, “other”, respectively). Sex was categorised into three groups (“men”, “women”, “diverse”).

### Statistical Analyses

All data analysis was conducted using IBM SPSS Version 26. The association between outcome expectancies, risk perceptions, self‐efficacy, and intention was analysed using a standard multiple regression analysis with dummy‐coded control variables for hospital, time period, sex, age, and profession. For age, “younger than or 25 years old” was chosen as the reference group and compared to “26 to 40 years old” and “41 years old or older”. Concerning profession, “physicians” were used as the reference group and compared to “midwives”, “nurses”, and “other”. A multiple mediation analysis examining the association between intention and hand hygiene behaviour was conducted with bootstrap analyses using Process macro for SPSS version 3.4. Time period, hospital, sex, age, and profession were added as dummy‐coded covariates and thus adjusted for in all independent and mediator variables (Hayes, 2012). Coping self‐efficacy, coping planning, and perceived resources were added as mediators.

### Ethical Approval

Ethical approval was granted from both hospitals and the Ethics Committee at Jacobs University Bremen (dated 17 September 2019). All study participants provided written informed consent to participate in the study. Data security did not allow for correlations between observed and self‐reported data.

## RESULTS

### Adherence to WHO Hand Hygiene Recommendations over Time and Hospitals

#### Observations

In total, 267 moments to wash or sanitise hands according to the WHO standards were observed in both hospitals, including 75 (28%) *before touching a patient* and 91 (34%) *after touching a patient*, 39 (15%) *before aseptic procedures*, 37 (14%) *a* *fter touching patient surroundings*, and 25 (9%) *after body fluid exposure risk*. We observed nurses in 95 (36%), midwives in 73 (27%), and physicians in 64 (24%) moments. For some moments, midwives in training (*n *=* *20, 7%) and physicians in training (*n *=* *7, 3%) were observed. Finally, a few observations regarded physiotherapists (*n *=* *8, 3%). Observations were made either in the delivery rooms (*n *=* *163, 61%) or postpartum units (*n *=* *104, 39%).

**FIGURE 1:**
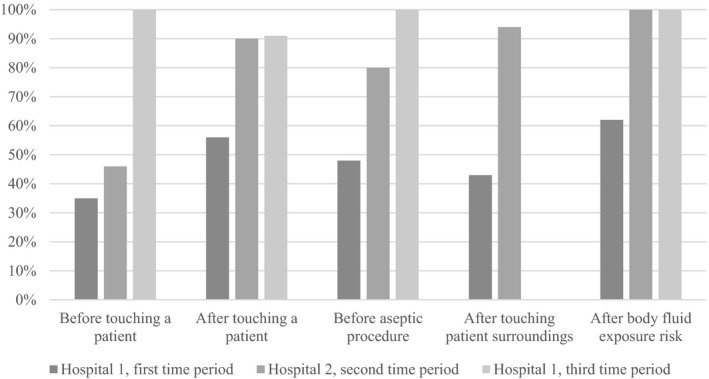
Observed adherence to hand hygiene recommendations over time. *Note*: The figure shows 148 *observations* from the first hospital during the *pre‐COVID‐19 pandemic period*, 98 observations from the second hospital in the *heightened awareness period*, and 21 observations from the first hospital in the last time period (*strict precautions*). Due to the low number of moments, the last period (*strict precautions*) needs to be interpreted with caution. There were no observed moments concerning *after touching patient surroundings* due to the lockdown on 16 March.

During the first episode (*pre‐COVID‐19 pandemic*), HCW in the first hospital had an overall adherence of 47 per cent (70 of 148), ranging from 35 per cent concerning *after touching a patient* to 62 per cent *after body fluid exposure risk*. In the second hospital during the *heightened awareness period*, observations showed an overall adherence rate of 79 per cent (77 of 98). The lowest adherence was found for the moment *before touching a patient* with 46 per cent, the highest adherence was seen *after body fluid exposure risk* (100%). In the last period (*strict precautions*), observations can only be reported for total adherence following the WHO recommendations to interpret hand hygiene observations.^1^ HCW adhered to hand hygiene recommendations in 20 of 21 moments, leading to an adherence rate of 95 per cent. An overview over all moments is provided in Figure 1.

Adherence to hand hygiene recommendations differed significantly between the first and second hospital/time periods with adherence being higher in the second hospital/time period (χ^2^(*df* = 2) = 35.02, *p* < .001). Adherence to recommendations did not differ significantly between occupational groups (χ^2^(*df* = 5) = 4.2, *p* < .52).

#### Self‐Reported Hand Hygiene Behaviour

Healthcare workers from both hospitals reported good hand hygiene behaviour during the first two time periods (*M* = 5.03, *SD* = 0.75). The highest adherence to recommendations was reported for the moment *after body fluid exposure risk* (*M* = 5.66, *SD* = 0.67). Lowest adherence was reported *after touching patient surroundings* (*M* = 4.5, *SD* = 1.26); other moments were *before touching a patient* (*M* = 4.63, *SD* = 1.04), *before aseptic procedures* (*M* = 5.16, *SD* = 1.04) and *after touching a patient* (*M* = 5.17, *SD* = 0.87).

### Social‐Cognitive Determinants of Hand Hygiene Behaviour

To test the association between outcome expectancies, risk perceptions, and self‐efficacy with intention to wash or sanitise hands, a multiple regression was calculated (adjusted *R* ^2^ = .267). Controlling for hospital, time period, sex, age, and profession, only self‐efficacy (*β *= .469, *p* < .001) was associated positively with the intention to wash or sanitise hands. Outcome expectancies and risk perceptions were not associated significantly with intention. The regression analysis showed gender differences in so far as women reported better adherence (*β *= −.255, *p *= .022). 38.4 per cent of the variance of intention could be explained (*R* ^2^ = .384). Post‐hoc power analyses again showed an adequate power only for medium‐to‐large effect sizes (*1 – β *= .882, *f* ^2^ **= .2). Results are shown in Table 2.

**TABLE 2:** Multiple Regression Analysis on Intention to Wash or Sanitise Hands According to WHO Recommendations

	*Unstandardised coefficients*		*Standardised coefficients*	*t*	*p (two‐tailed)*
	*B*	*SE*	*β*		
Intercept	3.13	.67		4.71	<.001
Self‐efficacy	.25	.06	.47	4.03	<.001
Risk perceptions	.04	.04	.09	.90	.371
Positive outcome expectancies	.11	.11	.11	1.02	.314
Negative outcome expectancies	.05	.06	.11	.98	.330
Hospital^a^	.16	.15	.14	1.08	.286
Time period^a^	.04	.14	.03	.31	.756
Sex^a^	−.52	.22	−.26	−2.35	.022
Age 1^b^	−.10	.18	−.08	−.54	.589
Age 2^b^	−.22	.21	−.15	−1.04	.302
Profession 1^c^	.07	.15	.06	.46	.646
Profession 2^c^	.22	.22	.13	1.01	.318
Profession 3^c^	−.01	.26	−.01	−.05	.962

Note

Self‐reported data were collected in two hospitals simultaneously over two time periods. The table does not show repeated measures/panel data.

^a^ Hospital, time period and sex (0 = “woman”, 1 = “man”) were added as dummy‐coded control variables.

^b^ For age, “younger than or 25 years old” was chosen as reference group and compared to “26–40 years old” (contrast age 1) and “41 years old or older” (contrast age 2).

^c^ Concerning profession, “physicians” were used as the reference group and compared to “midwives” (contrast profession 1), “nurses” (contrast profession 2), and “other” (contrast profession 3).

**FIGURE 2:**
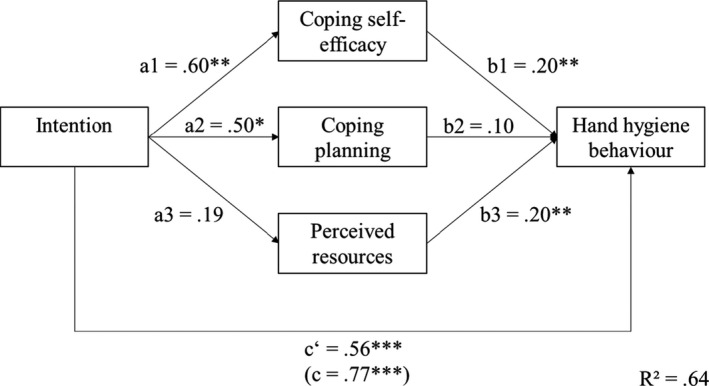
Mediation analysis between intention and hand hygiene behaviour. *Note*: The figure shows the multiple mediation analyses between *self‐reported* intention to wash or sanitise hands and handwashing behaviour. The figure does not show repeated measures panel data. Time period, hospital, sex, age, and profession were controlled for using dummy‐coded covariates for all variables. * significant at the .05 α‐level; ** significant at the .01 α‐level; *** significant at the .001 α‐level.

A multiple mediation analysis was conducted to test the association between intention and hand hygiene behaviour when controlling for hospital, time period, sex, age, and profession. HCW who were 25 years of age or younger reported slightly better hand hygiene than HCW who were between 26 and 40 years old (*β *= .406, *p* < .001). The analysis revealed a total standardised effect (*c *= .596, *p* < .001) as well as a smaller direct standardised effect (*cʹ *= .432, *p* < .001) between intention and hand hygiene behaviour. Intention had significant associations with coping self‐efficacy (*α *=* *.327, *p* = .002) and coping planning (*α *= .254, *p* = .023), but not with perceived resources (*α *= .119, *p* = .264). Further, coping self‐efficacy (*β *= .294, *p *= .002) and perceived resources (*β *= .250, *p *= .006) were significantly associated with hand hygiene behaviour in the mediation model (Figure 2).

Bootstrap analyses showed a significant standardised indirect effect for coping self‐efficacy (α*β = .096, 95% CI [0.02, 0.199]) and a significant standardised total indirect effect (α*β = .164, 95% CI [0.049, 0.312]). Overall, 62.6 per cent (*R* ^2^ = .626) of variance could be explained.

## DISCUSSION

In this study, we aimed to investigate hand hygiene behaviour in obstetric HCW during the COVID‐19 pandemic. The first objective was to examine to what extent the COVID‐19 pandemic related to a hypothesised increase in adherence to hand hygiene recommendations among obstetricians and midwives. We observed 267 moments to wash or sanitise hands according to the WHO observation recommendations in three time periods (*pre‐COVID‐19 pandemic*,*heightened awareness*, and *of strict precautions*). The results support the assumption that the COVID‐19 pandemic has affected hand hygiene behaviour. However, we were unable to observe hand hygiene behaviour in both hospitals during all time points. Therefore, differences in adherence at baseline may have influenced the observed adherence. The second aim of this study was to determine social‐cognitive factors that are associated with self‐reported hand hygiene behaviour. The findings support the theoretical predictions in part. To see if self‐reported adherence was accurate, we compared self‐reported and observed hand hygiene behaviour in the same HCW and detected discrepancies.

In further detail, observations revealed that adherence to hand hygiene in the first hospital during the *pre‐COVID‐19 period* (before the first official registered case in Germany on 28 January) was 47 per cent. This is slightly above the global average of 38.7 per cent in 2009 put forward by the WHO when they warned of low compliance worldwide based on more than 1,100 reviewed sources (WHO Patient Safety Alliance, 2009). Since then, many studies have focused on improving hand hygiene adherence. Interventions have increased adherence to 50–80 per cent using different methodologies such as automated feedback or video cameras. However, the ideal monitoring or intervention method has not yet been found and improper hand hygiene continues to be a problem (Boyce, 2019). In accordance with the available literature (Alshammari et al., 2018; Jenner et al., 2006), observed hand hygiene adherence was lower than self‐reported adherence in this study. The initial high levels of self‐reported adherence could reflect social desirability or a self‐serving bias (Contzen, Pasquale, & Mosler, 2015; Foà et al., 2017). Thus, our results support earlier findings that self‐reported hand hygiene behaviour needs to be assessed as objectively as possible (WHO Patient Safety Alliance, 2009). Seeing that adherence to hand hygiene recommendations has been found to be lower in obstetrics and gynaecology (Cantrell et al., 2009; Santosaningsih et al., 2017), the adherence we found in the baseline period is relatively good. However, there is still a clear need for improvement to meet the recommended target adherence level of 100 per cent to avoid HAI (WHO Patient Safety Alliance, 2009).

It seems that HCW adapted their observable behaviour after the outbreak of COVID‐19 in Germany. In the *period of heightened awareness*, observed adherence in the second hospital was 79 per cent. Our χ^2^‐test revealed that this was significantly higher than hand hygiene adherence in the *pre‐COVID‐19 period* in the first hospital, which is in line with our first hypothesis. It must be kept in mind, however, that the baseline for the second hospital was not known so we cannot construe the higher adherence as being an increase due to the COVID‐19 pandemic without alternative explanations. The effect might have been due to baseline differences or general measures in the clinics such as setting up dispensers and spreading information material. In the short *period of strict precautions* just before lockdown on 16 March, standard operational procedures regarding COVID‐19 were implemented, including testing and isolating patients, wearing face masks at all times, and taking special precautions during delivery. In this period, only a small number of observations could be made (*n *=* *21 with five different HCW) due to the precautions. Our observations revealed an exceptionally high adherence of 95 per cent. Thus, observed adherence to hand hygiene recommendations seems to have improved after the outbreak of the COVID‐19 pandemic in Germany as we expected in our first hypothesis. This is in line with other preliminary findings examining hand hygiene during the COVID‐19 pandemic (Israel et al., in press), suggesting that HCW adapted well to new requirements. This increase in hand hygiene adherence could prove to be a unique chance to improve hand hygiene in the future since positive habits can be formed (Diefenbacher et al., 2020).

A possible psychological explanation is that HCW experienced a higher need to prevent infections specifically caused by the SARS‐CoV‐2 virus. Fear of infecting oneself or one’s family has been found to be associated with better adherence among HCW (Houghton et al., 2020) and to drive public health responses (Harper et al., 2020). In terms of the HAPA model, fear and the perceived need to prevent infections translate to risk perceptions, outcome expectancies, and intention. However, a behavioural intention can only translate into better hand hygiene behaviour when HCW also feel confident to adapt. A number of barriers can affect their self‐efficacy, including constantly changing policies, high workload, and insufficient space to isolate patients. The COVID‐19 pandemic has been characterised by a rapid progression and accordingly changing guidelines. Consequently, barriers that could threaten the quick adaptation of hand hygiene behaviour need to be addressed. An adequate workplace culture, training, and support from managers as well as addressing knowledge and decision‐making processes is needed (Houghton et al., 2020; Srigley et al., 2015).

To test the determinants of observed behaviour, we investigated the link between social‐cognitive factors and hand hygiene behaviour in light of the HAPA model (Schwarzer, 2008) in our second hypothesis. In the regression model, only self‐efficacy and sex were significant predictors when we controlled for hospital, time period, age, and profession. Higher adherence among women has been described before and is thus not surprising (Suen et al., 2019). The results only partly fit our hypothesis, since risk perceptions and outcome expectancies were not associated significantly with intention. In non‐clinical samples, outcome expectancies were found to be associated with the intention to wash or sanitise hands which stands in contrast to our results in a clinical setting (Reyes Fernández et al., 2016). Self‐efficacy showed a strong association with the intention to wash or sanitise hands. This stands in agreement with prior research on the HAPA model (Lhakhang et al., 2015; Schwarzer et al., 2011). Probably all HCW in our study perceived a high need for adequate hand hygiene, but only those with high self‐efficacy seemed to have formed the intention to change their behaviour.

The second part of the second hypothesis focused on actual hand hygiene behaviour. A multiple mediation analysis was conducted to test for mediation effects of coping self‐efficacy, coping planning, and perceived barriers between the intention to wash or sanitise hands and hand hygiene behaviour. Only coping self‐efficacy was a significant mediator. This emphasises its role for hand hygiene behaviour and shows how crucial self‐efficacy is for behaviour change in general (Lhakhang et al., 2015; Lippke et al., 2010). In behaviour change theories, the “intention–behaviour gap” means that the decision to change behaviour does not necessarily lead to action. This gap can be bridged by planning and maintenance/ coping self‐efficacy (Sniehotta et al., 2005). Although perceived resources (availability of dispensers, training, and social support from superiors) were positively associated with hand hygiene behaviour, they did not *mediate* the effect from intention to actual behaviour. This result is still in compliance with the literature which has shown that perceived and objective resources can promote hand hygiene unrelated to prior intention (Sadule‐Rios & Aguilera, 2017). Higher adherence could thus be achieved by addressing barriers such as time constraints and the unavailability of resources (Pittet et al., 2017). In the mediation model, age was also significantly associated with hand hygiene behaviour. HCW between the age of 26 and 40 reported better hand hygiene behaviour than those under the age of 26. It is possible that these HCW have formed a greater routine and thus a better habit in sanitising hands. Diefenbacher et al. have confirmed that habits are crucial for maintaining good hand hygiene (Diefenbacher et al., 2020).

Our study has several limitations as well as some implications for future research. Most importantly, we could not conduct observations in both hospitals during all three time periods but only in the first hospital for the first (*pre‐COVID‐19 pandemic*) and third (*strict precautions*) time periods, while the observations during the second time period (*heightened awareness*) were conducted in the second hospital. It is possible that the baseline adherence to hand hygiene recommendations was already higher in the second hospital before the *heightened awareness period*, so we cannot conclude that there was actually an increase in adherence. Only self‐reported data were not confounded as they were collected at both hospitals for both time periods. Future research should conduct observations in a longitudinal design at multiple sites, especially during a possible second wave of the COVID‐19 pandemic. During the third time period, only a very small number of handwashing moments (*n *=* *21) could be observed in a small number of participants (*n *=* *5). Thus, it is not possible to compare adherence rates from this period with the *pre‐COVID‐19 pandemic* beyond description. More observations during the period of *strict precautions* would have been valuable for conclusions but, practically, it was not feasible. On the descriptive level, adherence improved since all HCW in this period showed nearly perfect adherence. Nevertheless, this conclusion should be drawn with caution considering that we might have unconsciously observed more adherent HCW again in the third period. However, the results speak for a general increase in hand hygiene due to the COVID‐19 pandemic. Secondly, we did not directly assess any specific changes in self‐reported hand hygiene adherence and social‐cognitive factors regarding the COVID‐19 pandemic. When we started data acquisition, the COVID‐19 pandemic had not reached Germany. Most of the HCW had already answered the questionnaire when strict precautions started so we refrained from adding a direct question regarding perceived changes of HCW’s hand hygiene. Further research should aim to account for changes over time within self‐reported data directly. Thirdly, we have a hierarchical data structure which we aimed to control for by using dummy‐coded variables. Using hierarchical linear modelling might be more accurate for data from multiple hospitals but was not applicable due to the small number of level‐2 units (hospitals). Future studies could aim for more recruitment sites. Finally, some methodological issues emerged due to practical and data security requirements. We used behavioural observations to generate more reliable data on hand hygiene adherence during the beginning of the COVID‐19 pandemic. However, these data cannot be integrated statistically with the self‐reported data since we cannot match participants. We did not assess any correlation between observed and self‐reported data which would be valuable in further research. Since HCW were often working under time pressure, we needed to use single‐item scales to ensure acceptability. Single‐item scales need to be treated with caution because they might not be appropriate for heterogeneous constructs and have a lower reliability. For the same reason, we also did not assess action planning. We assumed that behavioural recommendations for hand hygiene behaviour were quite specific. We instead focused on coping planning. However, action planning is an important construct from the HAPA model and should be considered in the future (Schwarzer et al., 2011). Age needed to be assessed in categories to ensure anonymity although every categorisation causes a loss of information. In the analyses regarding self‐reported data, we aimed to control for age which would have been more accurate with a continuous variable. Since age and profession needed to be added to the analyses as multiple dummy‐coded covariates, the number of predictors increased. Due to this increase and the small sample size of HCW at the two hospitals, only medium‐to‐large effect sizes could be detected. For mediation analyses, the power also depends largely on effect sizes (Schoemann et al., 2017). Thus, smaller but still important mediation effects (e.g. for coping planning) might not have been found and need to be re‐examined with a higher and previously planned sample size. In addition, we used mediation analyses on cross‐sectional data which violates model assumptions. Since longitudinal processes cannot be depicted using data from only one time point, the behaviour change over time might be more complex than we found in our cross‐sectional model. Behaviour change regarding hand hygiene should be examined in a longitudinal design and with observed hand hygiene adherence to ensure validity.

Nevertheless, this study is to our knowledge the first to examine hand hygiene behaviour in HCW during the COVID‐19 pandemic while simultaneously investigating social‐cognitive determinants. We had a theory‐based approach in two sites using self‐reported data as well as more objective, unobtrusive observations so that results can be generalised to other obstetric university hospitals. Our results indicate that hand hygiene behaviour in two obstetric hospitals improved in the face of a pandemic threat. At this time, only low‐cost interventions were used such as posters about COVID‐19, more sanitiser dispensers, and finally issuing operational procedures. Future research should examine the underlying psychological processes of the increase in adherence to hand hygiene recommendations. As the HAPA model was applicable to this behaviour even during the pandemic, interventions should make use of the theory and its assumptions to ensure evidence‐based interventions towards patient safety. In particular, (coping) self‐efficacy should be addressed in future interventions. Another interesting aspect is the effect of gradually terminating COVID‐19 regulations issued by local health authorities. It would be valuable to see whether adherence to hand hygiene recommendations remains on a high level or if it drops back to its initial baseline. The pandemic could prove to be a unique chance to improve hand hygiene in the long term, so research should focus on how to maintain high hand hygiene adherence after the COVID‐19 pandemic. Interventions should be theory‐driven, tailored to the individual, and consider intention and coping self‐efficacy. Future interventions should also provide resources such as sanitiser availability and support from superiors as well as organisational culture and psychological barriers relating to coping self‐efficacy.

## Supporting information


**Table S1.** Overview over data collection.
**Appendix S1.** Translated observer sheet.

## Footnotes

^1^
According to the WHO, at least 20 observations should be conducted to interpret single indications. Due to the lockdown, this was not possible in the current study.
