# Supplementary material for: Obstetric Healthcare Workers’ Adherence to Hand Hygiene Recommendations during the COVID‐19 Pandemic: Observations and Social‐Cognitive Determinants
Source: Appl Psychol Health Well Being. 2020 Oct 5;12(4):1286–305. doi: 10.1111/aphw.12240 (PMC7675238; doi:10.1111/aphw.12240)
Supplement: Supplementary file 1 — Table S1. Overview over data collection. Appendix S1. Translated observer sheet. [file APHW-12-1286-s001.docx]

**Appendix**

**Table A1**

Overview over data collection.

|  |  | *Pre-COVID-19 period* | *Period of heightened awareness* | *Period of strict precautions* |
| --- | --- | --- | --- | --- |
| Number of observations (number of observed participants) | First hospital | 148 (28) | 0 (0) | 21 (5) |
|  | Second hospital | 0 (0) | 98 (17) | 0 (0) |
| Questionnaire data^1^ | First hospital | 34 | 14 | 0 |
|  | Second hospital | 43 | 14 | 0 |

*Note*: Numbers are provided as total numbers per hospital and time period. ^1^ 5 participants from the first hospital and 5 participants from the second hospital did not indicate when they completed the questionnaires.

**Appendix S1.** Translated observer sheet


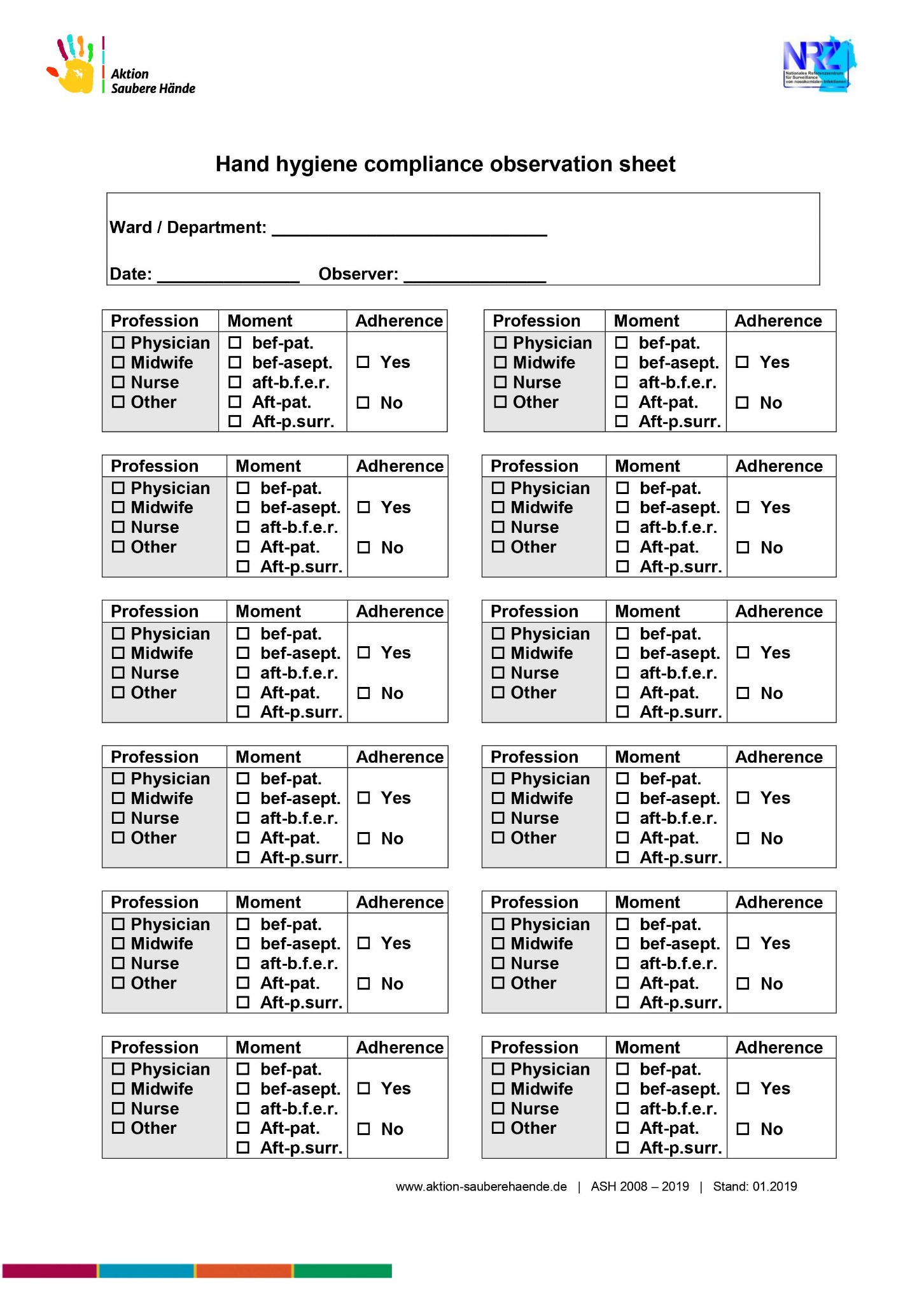


*Note*: The observer sheet used in this study was the German original. It can be found under the link https://www.aktion-sauberehaende.de/fileadmin/ash/user_upload/pdf/messmethoden

/1_Anleitung_zur_Beobachtung_Januar_2017.pdf

The observer was asked to code whether the observed HCW is a physician, nurse or “other” which was expanded by the category “midwife” to adapt the observation sheet to our obstetric setting. The sheets provided an overview of the five moments to wash or disinfect your hands (*before* and *after touching a patient*, *before aseptic procedures*, *after touching patient surroundings* and *after body fluid exposure risk*). The observers marked which moment they observed and whether the observed person adhered to the standards (i.e. whether they washed and/ or disinfected their hands). According to the recommendations, each moment should be observed at least 20 times for analysis. Observations were conducted by a research associate and two study nurses who were trained with the information material provided by “Clean Hands Campaign” (Aktion Saubere Hände, 2017).

Source: Aktion Saubere Hände. (01/2017). Anleitung zur Beobachtung der Händedesinfektion. https://www.aktion-sauberehaende.de/fileadmin/ash/user_upload

/pdf/messmethoden/1_Anleitung_zur_Beobachtung_Januar_2017.pdf
